# Differential phase contrast for quantitative imaging and spectro-microscopy at a nanoprobe beamline

**DOI:** 10.1107/S1600577522010633

**Published:** 2023-01-01

**Authors:** Paul D. Quinn, Fernando Cacho-Nerin, Miguel A. Gomez-Gonzalez, Julia E. Parker, Timothy Poon, Jessica M. Walker

**Affiliations:** a Diamond Light Source, Harwell Science and Innovation Campus, Didcot, Oxfordshire OX11 0DE, United Kingdom; University of Malaga, Spain

**Keywords:** differential phase contrast, DPC, ptychography, nanoprobe, spectro-microscopy

## Abstract

The interaction of a focused X-ray beam with a sample in a scanning probe experiment can provide a variety of information about the interaction volume. In many scanning probe experiments X-ray fluorescence (XRF) is supplemented with measurements of the transmitted or scattered intensity using a pixelated detector. The automated extraction of different signals from an area pixelated detector is described, in particular the methodology for extracting differential phase contrast (DPC) is demonstrated and different processing methods are compared across a range of samples. The phase shift of the transmitted X-ray beam by the sample, extracted from DPC, is also compared with ptychography measurements to provide a qualitative and quantitative comparison. While ptychography produces a superior image, DPC can offer a simple, flexible method for phase contrast imaging which can provide fast results and feedback during an experiment; furthermore, for many science problems, such as registration of XRF in a lighter matrix, DPC can provide sufficient information to meet the experimental aims. As the DPC technique is a quantitative measurement, it can be expanded to spectroscopic studies and a demonstration of DPC for spectro-microscopy measurements is presented. Where ptychography can separate the absorption and phase shifts by the sample, quantitative interpretation of a DPC image or spectro-microscopy signal can only be performed directly when absorption is negligible or where the absorption contribution is known and the contributions can be fitted.

## Abstract 2

Quantitative differential phase contrast using a pixelated detector is described and results for a range of samples are presented. The results are compared with ptychography and the application of differential phase contrast for spectro-microscopy is explored.

## 1. Introduction

Scanning X-ray microscopes use focused X-ray beams to measure local variations in composition, structure and morphology. The sample is raster-scanned through the focused beam and multiple signals or measurements can be collected at each point. In the hard X-ray regime techniques such as X-ray fluorescence (XRF) can record elemental compositions but are typically limited to higher atomic numbers (*Z* > 14) due to air paths between the sample and detector and the XRF detector window material. Measurements of absorption are similarly sensitive to higher atomic numbers and higher densities. Phase contrast imaging provides a mechanism to increase the sensitivity to lighter elements, by exploiting the fact that the real part of the refractive index, δ, is much larger than the imaginary part or absorption, β, and measures the resulting shifts in the X-ray phase which occur due to interaction with the sample (Pfeiffer *et al.*, 2006 ▸). The measured phase shift of the transmitted beam by the sample, ϕ, is related to the index of refraction, δ, and sample thickness, *t*, by

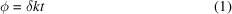

where *k* = 2π/λ.

From the phase shift, when measured far from the resonance energy, quantitative details of the composition or mass density of the sample and low-*Z* composition can be extracted given the formula for the index of refraction, δ,

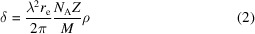

where λ is the X-ray wavelength, *r*_e_ is the classical electron radius, *N*_A_ is Avogadro’s constant, *Z* and *M* are the number of electrons (atomic number) and molecular weight of the sample, respectively, and ρ is the mass density (Giewekemeyer *et al.*, 2010 ▸; Schwenke *et al.*, 2021 ▸).

While phase contrast can provide important information by itself, when coupled with XRF it can provide an understanding of how heavier elements are dispersed in a light-element structure within the sample. For example, an investigation of the use of targeted metallo-drugs in biological soft tissue requires an identification of the metal species in the cell and the location of those metal species within the context of the cell structure (Deng *et al.*, 2018 ▸). Phase contrast imaging has also been used as a supporting measurement to guide registration for XRF tomography (Hong *et al.*, 2015 ▸)

X-ray ptychography has advanced as a method to obtain high-resolution images of the absorption and phase shift of the transmitted beam by the sample (Yu *et al.*, 2018 ▸; Wise *et al.*, 2016 ▸). 2D coherent scattering patterns are collected by scanning the sample at sequential overlapping regions, and an iterative phase-retrieval algorithm is used to retrieve the phase information from the recorded intensities (Rodenburg *et al.*, 2007 ▸; Maiden & Rodenburg, 2009 ▸).

For multimodal experiments, correlating XRF with ptychography can present some challenges. Ptychography measurements are typically performed out of focus with beams of 1–10 µm but the resolution of any XRF image acquired will be limited by the probe size. The XRF resolution would then have to be retrieved using deconvolution approaches (Vine *et al.*, 2012 ▸). Alternatively focused beam measurements can be used for ptychography allowing XRF to be acquired simultaneously. However, the overlap requirement for ptychography can increase the number of sampling points beyond that of a typical XRF scanning probe measurement, increasing data volume, collection and processing times. Addressing the computational requirements of ptychography to deliver fast and routine operation is an ongoing area of development across facilities (Nashed *et al.*, 2017 ▸; Cherukara *et al.*, 2020 ▸; Wakonig *et al.*, 2020 ▸).

In contrast to ptychography, differential phase contrast (DPC) imaging produces an image of the phase shifts to the X-ray beam as a result of interaction with the sample by measuring the gradient of the phase and retrieving the phase shifts by a straightforward integration step.

The DPC method is used across optical, electron and X-ray systems, and can be implemented using either full-field or scanning probe approaches, although the method of extraction of the differential signal differs across the techniques (Shibata *et al.*, 2012 ▸; Liu *et al.*, 2013 ▸; Waddell & Chapman, 1979 ▸). In the X-ray regime the scanning DPC technique has previously been implemented using charge-coupled detectors (Chapman *et al.*, 1996 ▸) but was limited in its broader application by readout times and signal to noise. Segmented detectors allowed for the differential signal to be extracted for quantitative measurements at a high rate (Hornberger *et al.*, 2007 ▸), but with recent advances in direct detection pixelated detectors it is now possible to measure the beam interaction and resulting scattering or diffraction pattern at high frame rates and extract a range of information (Bunk *et al.*, 2009 ▸; Krajnak *et al.*, 2016 ▸). For nano-focusing experiments this extends the information content in multimodal experiments as DPC can be performed at focus and simultaneously with XRF without compromising detection limits, and with relaxed conditions regarding acquisition step size compared with ptychography. However, unlike ptychography, the resolution of the reconstructed image is probe size limited

Here, we describe the methodologies for measuring the intensity and phase as implemented for routine operation on the hard X-ray nanoprobe beamline, I14, at Diamond Light Source (Quinn *et al.*, 2021 ▸). The phase integration step is discussed, and a quantitative comparison is made with ptychography. The application of the DPC technique to hard X-ray spectro-microscopy is also examined.

## 2. Intensity and DPC imaging

When a beam probe interacts with a sample the beam can be absorbed, the beam will be refracted, and diffraction can occur from the interaction with the sample. The absorption and refraction of the beam and the scatter or diffraction pattern from the interaction volume will be recorded as an intensity distribution on the detector. Descriptions of the intensity on the detector and the different contributions to the signal have been described in detail elsewhere (De Jonge *et al.*, 2008 ▸; Thibault *et al.*, 2009 ▸; Waddell & Chapman, 1979 ▸). To measure the phase shift contribution from the sample the deflection angle of the beam in each direction, α_*x*_ and α_*y*_, is related to the phase gradient by

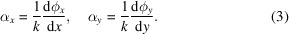

The phase shift, Φ, can then be obtained, by integration, from the phase gradients in each direction.

From the intensity on the detector, in addition to the deflection from the phase gradient, the absorption signal can be extracted, and the dark-field signal can be extracted from the intensity on the detector in the absence of the beam probe, *i.e.* the scatter signal. The dark-field or scattering signal represents the contrast arising from sample inhomogeneities within the volume sampled by the beam. The different signals, absorption, DPC and dark-field, can also be considered as different moments of the detected signal (Modregger *et al.*, 2017 ▸) and while the dark-field signal can be defined as the second moment, or variance, in the scattering distribution, this was not used here and only the sum or gradient of different signals extracted from the detector were considered.

## 3. Extracting intensity and differential contrast signals

The DPC measurements presented here were measured on the nanoprobe beamline at Diamond Light Source (Quinn *et al.*, 2021 ▸) using a quad Medipix (Ballabriga *et al.*, 2011 ▸) Merlin detector (4× Medipix3, 55 µm pitch, 512 × 512 pixels, Quantum Detectors, UK) which was placed 1.8 to 2.1 m from the sample. The beamline uses nano-focusing mirrors, achieving a 50 nm focus, with the resulting beam projection on the detector resembling diffraction from a rectangular aperture, in comparison with the circular apertured beam from previous DPC studies using zone plate systems. The methodologies discussed below are generally applicable but have only been tested for the case of a rectangular beam.

To extract different signals from the measured intensity the detector image needs to be masked appropriately. The process of creating a mask was automated to remove the need to readjust for small changes in the Kirkpatrick–Baez mirrors or detector positions over a user run period. The automated masking uses an Otsu thresholding method (Otsu, 1979 ▸) to differentiate the transmitted beam intensity from the weaker scattering or diffraction signal and this allows us to selectively mask the beam region, or the mask can be scaled as required to define a smaller or larger region around the beam. Dead pixels, hot pixels and underperforming pixels are removed by an examination of the standard deviation of pixel intensities across the detector and a combination of a Hampel filter (Davies & Gather, 1993 ▸) and median filters. Detector sensor gaps at known positions are also included in the mask.

For processing, the sum of the pixels in the masked regions and the centre of mass (COM) of those pixels are recorded to provide different measures of the intensity and deflections of the beam and scatter. For DPC the scatter represents contributions from objects much smaller than the beam and so the region around the beam on the detector should be tightly cropped for the COM estimated to minimize the scatter contribution.

The integration of the DPC signal from the COM values, dΦ_*x*_ and dΦ_*y*_, is typically performed using surface normal integration approaches developed for the shape-from-shading problem. A number of different solutions based on the calculus of variations (Horn & Brooks, 1986 ▸), direct line integration (Wu & Li, 1988 ▸) or Fourier integration have been developed (Frankot & Chellappa, 1988 ▸; Simchony *et al.*, 1990 ▸). The Fourier approaches are efficient, have been developed to address potential problems of integrability with the added benefit that Fourier transforms, and their manipulation, are generally familiar to imaging scientists so this approach has been adopted here. The integration of the phase shift, Φ, can be written as

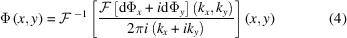

where (*k_x_*, *k_y_*) represent the reciprocal-space coordinates corresponding to (*x*, *y*) (Kottler *et al.*, 2007 ▸). This notation is commonly used but for quantification note that it is not the same as the wavevector, **k**, as there is no factor of 2π in the reciprocal coordinate. For phase integration, the argument of the inverse Fourier transform (4) is set equal to zero where *k* is zero. In practice, this means that the phase variation in the reconstruction is relative to an arbitrary constant and the resulting image, therefore, represents phase differences, not an absolute phase.

An issue that can affect phase integration is that sharp steps occur in the periodic boundary used in the fast Fourier transform (FFT). A common approach to solve this is to apply a mirror transform, *T*, about the edge in each direction, increasing the image size by 4, but providing an extended image of the gradients in each direction, dΦ_extended_ (5), without boundary discontinuities in the FFT (Mukaide *et al.*, 2009 ▸; Arnison *et al.*, 2004 ▸),

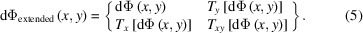

The FFT-based phase integration, (4), requires both continuity and differentiability of the phase gradient, and the mirroring approach can still result in such conditions. Imposing anti-symmetric boundary conditions has recently been proposed for optical measurements, and is adopted here, to address this by extending and mirroring the *x* direction phase gradient such that the sum along *y* is zero, and for the *y* gradient, the mirroring along *x* sums to zero (Bon *et al.*, 2012 ▸),

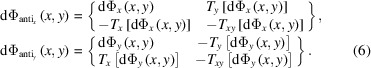

A notable alternative approach is to reformulate the problem to use discrete cosine transforms (DCT). A DCT is symmetric about its edges, by definition, although the result is in principle similar to the mirrored FFT result (Ishizuka & Ishizuka, 2020 ▸; Ishizuka *et al.*, 2017 ▸).

In some cases, the measurement and integration of the phase can still lead to low-frequency variations which reduce contrast. This can be addressed by alternatively formulating the integration step (Lazić *et al.*, 2016 ▸),

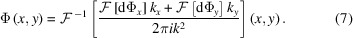

This can then be extended to introduce a tuneable scalar parameter, λ. This parameter will effectively suppress lower frequencies. This is also sometimes referred to as a form of Tikhonov regularization in the electron microscopy community (Piana & Bertero, 1996 ▸; Tian & Waller, 2015 ▸),

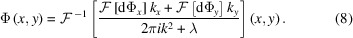

It should be clear that, while filtering may enhance some features, it will also impact on the quantification of the phase.

The determination of the beam deflection is performed by a conventional COM measurement on the image. FFT, thresholding and fitting methods (Yan *et al.*, 2013 ▸) were also investigated but, for our setup, they did not show comparable gradient sensitivity to the COM methods and the resulting integrated image generally required the use of high-pass filtering to achieve a representative image whereas the COM method generally did not require any additional filtering.

To demonstrate the automated masking, extracted signals and phase integration, experimental data were used from a selection of weakly and moderately absorbing samples. In general, isolated samples measured with uniform background and boundaries produce largely artefact-free DPC images and excellent examples can be found in the literature (Menzel *et al.*, 2010 ▸). The scans selected here have different edge boundaries and low-frequency artefacts which were useful to demonstrate the effects of the anti-symmetric mirroring and high-pass filter processing steps. The measurements were from (*a*) calcium carbonate plates (coccoliths) produced by coccolithophores, a marine algae (Walker *et al.*, 2020 ▸). Their small size (approximately 5 µm across with individual crystals of approximately 600 nm by 100 nm) and lower-*Z* element composition (mostly calcium carbonate with some other trace metals) contribute to their weak absorption. The measurements were performed at 12 keV using a 100 ms dwell time and 50 nm step size for the DPC and XRF. The dwell times used were dictated by the XRF signal intensity rather than the DPC, to simultaneously collect correlative XRF maps of the sample; measurements were also taken from (*b*) a Siemens star patterned from a 1 µm-thick tungsten film recorded at 12 keV using a 25 ms dwell time over 40 nm intervals, and (*c*) Fe powder (Fe_2_O_3_, Fe_3_O_4_ and FeO) mixed in a ball grinder and then drop-casted onto silicon nitride membranes. Maps were recorded at 8 keV with 80 nm step sizes and 15 ms dwell time per pixel.

## 4. Comparing intensity and phase images

**Figure 1:**
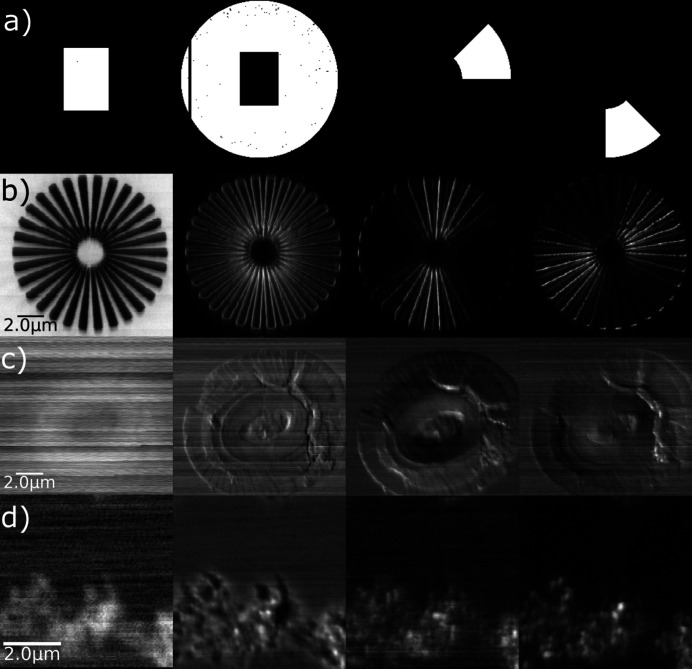
Intensity-based measurements extracted from the signal on the detector. (*a*) Left to right: masks for the extraction of absorption, scatter and example radially selected scatter intensities. (*b*)–(*d*) Left to right: corresponding absorption, scatter, and radially selected scatter intensities for (*b*) Siemens star, (*c*) coccolith, (*d*) drop-cast Fe particles.

Fig. 1 ▸ shows the automated beam selection, scatter (dark field) and radial masks used to select different signals along with examples of images from the intensity of the signal in these regions showing absorption, dark-field and radial dark-field signals which can be used to infer composition, orientation, and potentially size distributions under the beam probe. As expected, light-element samples such as the coccolith show weak absorption but the scattered signal, although weak, provides improved contrast and the directional effects of edges and interfaces in the scattering distribution can be seen in the radial scatter maps.

**Figure 2:**
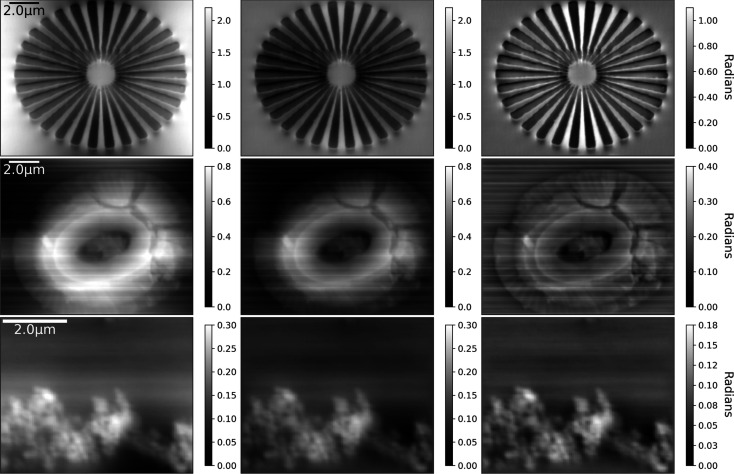
Left to right: DPC phase contrast image integrated using a mirror pattern, anti-symmetric pattern and high-pass filter (λ = 0.0005 times the maximum *k* ^2^ value) for measurements of a (top) Siemens star, (middle) coccolith and (bottom) drop-cast Fe particles. The anti-symmetric mirroring improves the low-frequency background. As the high-pass filter removes the low-frequency contributions, the mirror and anti-symmetric patterns have no impact on the resulting image.

Fig. 2 ▸ shows the results of phase integration of the COM measurements for the same set of samples. In this case, the mirroring (5) and anti-symmetric mirroring (6) are demonstrated for measurements of the beam deflection and the integrated phase, with high-pass filtering (8), is presented for comparison. The high-pass filtered image was largely indistinguishable between both the mirroring and anti-symmetric cases so only one example is shown for each case. The image contrast and background, or low-frequency components, are visibly improved using the anti-symmetric approach. In the weakly scattering coccolith sample, horizontal lines or bands of background variation, coming from artefacts in the phase gradient, are present. These band or phase ramp artefacts from the FFT integration are largely removed where the anti-symmetric approach is used and the background can be seen to be more uniform across the images. The high-pass filtering helps to improve the contrast of smaller features in all cases.

As already outlined, the phase measurement can be used to extract meaningful information on sample composition. While the diffraction pattern deflects with the phase gradient it should be clear that the COM is only an accurate measure of deflection when applied to the beam probe. The scattered or diffracted intensity will vary across the detector, often asymmetrically, depending on the sample interaction and this would therefore skew the COM, *i.e.* the phase is only quantitative for measurements of the COM of the beam when the scattered signal is masked out. A comparison between ptychography and DPC was performed on the coccolith sample to provide a practical comparison and explore the quantification of phase by DPC. Ptychography scans were collected 0.8 mm out of focus, 100 nm steps with 30 ms dwell time, in a continuous motion scan mode with ∼1.2 µm probe size at 9 keV and the ptychography data were processed using the *ptypy* package (Enders & Thibault, 2016 ▸). The focusing optic is coherently illuminated under these conditions. The difference map method (Elser, 2003 ▸) without position refinement with a single probe mode was used in the reconstruction.

**Figure 3:**
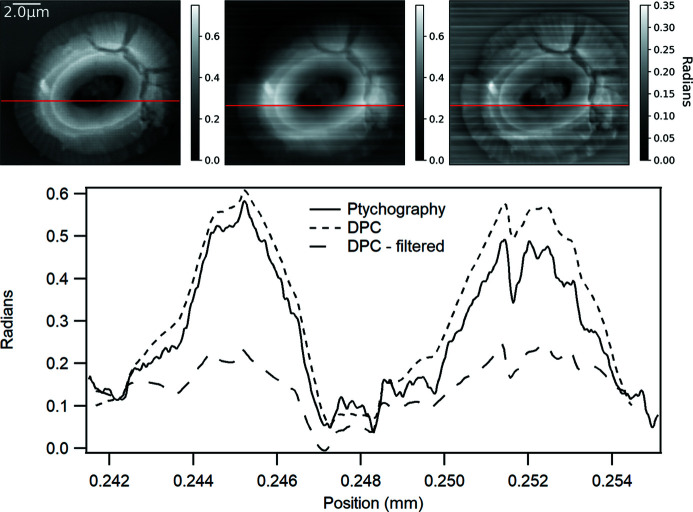
A comparison of DPC and ptychography from a coccolith sample. Top row left, ptychography phase image; middle, DPC phase image; right, DPC high-pass-filtered image. Bottom: comparison of phase line profile (indicated on phase images by a horizontal line) from DPC and ptychography. Note that the line profile from the ptychography has been offset to overlay with the start and end of the DPC line profile. No phase ramp removal was used on the images. The images are similar, albeit with stripes in the DPC, but the higher resolution of ptychography improves contrast in the depressions of the coccolith.

Fig. 3 ▸ compares the DPC integrated phase image from a coccolith with the corresponding ptychography phase image. A profile taken through the phase images, and offset to zero the background in both cases, shows good agreement, although with some differences due to a slight ramp over the image DPC. The resolution of the DPC was 50–60 nm as determined by the beam size and step size, and the ptychography was reconstructed to a 27 nm pixel size so the detail within recesses in the coccolith is smoothed as a result. Nevertheless, the phase quantification and image contrast compare reasonably well with ptychography. DPC does not require overlap of the probe position in a scan so can be used where the scan step may be equal to or larger than the probe size. The computational requirements of the COM and integration step are low which enables fast processing, limited only by data loading times when performed post-experiment. For *N* sample measurements, DPC requires *N* COM calculations and 2 FFTs, while a typical ptychography reconstruction requires roughly 2*N* FFTs per iteration with several 100 to 1000’s of iterations. The COM for each scan point can be calculated live as the scan progresses and the DPC calculated immediately at the end of the scan or even updated during the scan (Yu *et al.*, 2022 ▸) to provide images for decision making and feedback during an experiment. At-focus ptychography can also be processed as a DPC measurement to provide a complementary, but lightweight, pre-processing step to deliver phase images prior to a ptychography reconstruction step or to potentially seed a reconstruction (Wittwer *et al.*, 2022 ▸).

For weakly scattering signals the DPC process is, in our experience, very robust. While the automation of ptychography processing is improving, it can still require expert intervention and an iterative approach with various computational and optimization techniques to achieve the best results. Ptychography produces a higher-resolution, de-noised image but the low computational cost and ability to provide rapid results during an experiment make DPC a useful tool which may provide sufficient detail depending on the science needs of the experiment (Chevrier *et al.*, 2022 ▸).

### 4.1. Spectroscopy using differential contrast

The Kramers–Kronig (KK) relation connects the real and imaginary parts of the refractive index and thus provides a link between experimental absorption and phase measurements. X-ray absorption near-edge spectroscopy measurements (XANES) can therefore be used to study the chemical state of materials through measurement of the absorption (absorption or XRF), phase or both.

**Figure 4:**
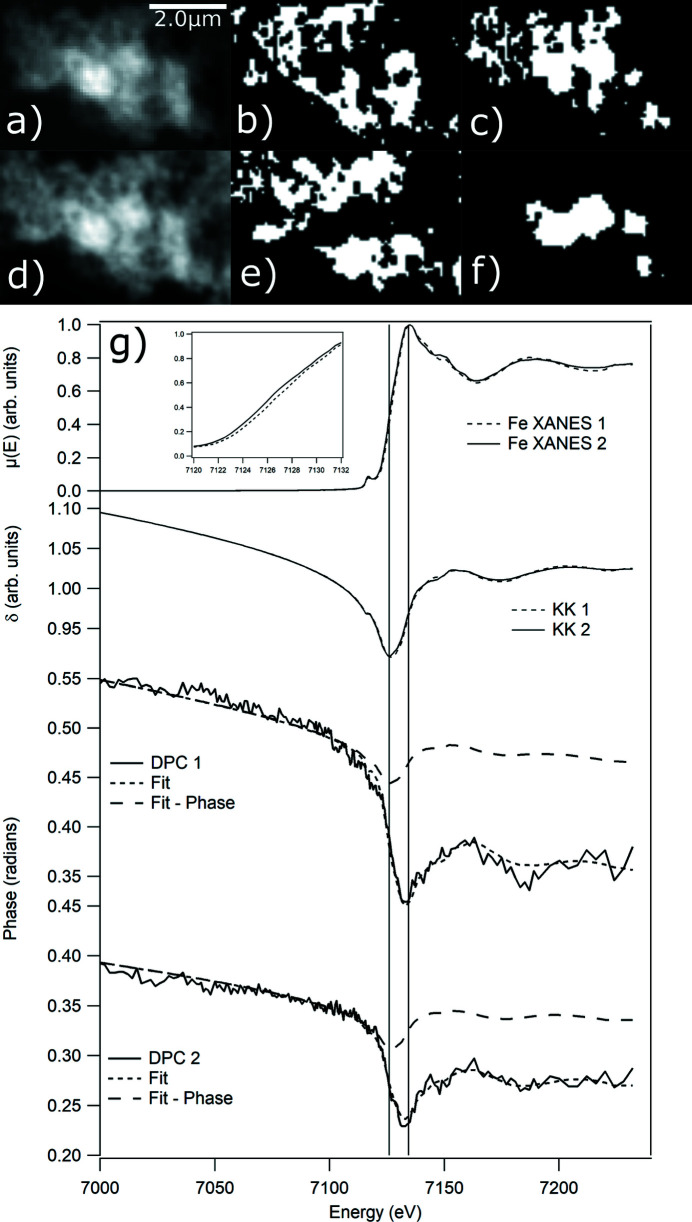
(*a*)–(*c*) DPC phase image and cluster labels from DPC-XANES signal of a Fe particulate sample. (*d*)–(*f*) XRF Fe image and cluster labels from XRF-XANES signal clustering. (*g*) (Top) XRF-XANES spectra from the two regions identified by DPC clustering and inset showing edge shift between spectra. (Middle) KK transform of the extracted Fe XRF-XANES. (Bottom) Extracted differential contrast signals from each cluster and linear combination fit of absorption and phase contributions to this signal. The fitted phase components are shown. The peak positions of XRF-XANES and KK transform are shown to demonstrate the peak of the differential signal lies between these two positions.

Differential contrast and XRF-XANES scans were taken on a region of the previously described Fe oxides mixture sample. 1 eV steps were used over the pre-edge to extract the pre-edge variations in δ. 0.5 eV steps were used from −20 eV to +30 eV around the Fe edge and a variable energy step was used after this. The phase images were processed, stacked, aligned and then clustered to extract the spectral components. The XRF-XANES spectra from the clusters identified using DPC were then averaged. Only three clusters were used for the study and the same processing steps were used in both studies. The XRF-XANES spectra were also clustered for comparison and an example of the phase image, XRF image, two extracted clusters from DPC and XRF, differential signals and XRF-XANES over the Fe edge from the two main clusters, excluding the background, are shown in Fig. 4 ▸. The clustered regions by XRF and DPC are different but broadly identify a similar central region

Interpretation of these measurements requires knowledge of the different contributions. Measurements made using DPC are a result of the combination of the differential absorption and differential phase contributions (De Jonge *et al.*, 2008 ▸; Thibault *et al.*, 2009 ▸). The differential absorption effect is often considered negligible in the literature. In many applications on a nanoprobe, ensuring negligible absorption is difficult with competing sample requirements for both the multimodal XRF or diffraction measurements and the underlying science problem. The reduction of beam sizes to the nanoscale also means absorption from the volume under the probe can make a significant contribution. The Fe sample studied had non-negligible absorption and the extracted DPC spectra in Fig. 4 ▸ show this mixture of phase and absorption contributions in the extracted signal. The absorption profile is visible in the differential signal but is shifted to lower energy and possesses a slowly varying pre-edge tail due to the contribution of the phase component.

The integrated differential signal, *g*, measured at energies, *E*, over the absorption edge can be approximated as a linear combination of the differential absorption and differential phase contributions with other contributions being linear over this range (Thibault *et al.*, 2009 ▸). The contributions, for the fit to the measured data, are represented here by

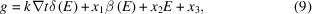

where *x*_2_ *E* and *x*_3_ represent a correction for linear variations and offset to the measured data over the range, respectively. The differential absorption changes the intensity profile on the detector and as a result the COM and the deflection measurement. The scaling factor, *x*_1_, is to account for the scaling from integrated phase measurement to the actual differential absorption.

The differential signal was fitted by a linear least-squares combination of the XRF-XANES signal and its KK transform signal (9). The KK transform of the XRF-XANES was determined using the *kkcalc* package (Watts, 2014 ▸). The results, Fig. 4 ▸, show the validity of the model for interpretation of the DPC-XANES with good agreement between the measured data and the fit. The result also demonstrates how non-negligible absorption can affect the interpretation of DPC results. Any measurement, in this case, at energies above the absorption edge will be offset by the differential absorption contribution and could therefore be misinterpreted if used as a quantitative measurement. Processing of the absorption signal, as outlined previously (Fig. 1 ▸), is an essential step to guide whether the DPC signal can be directly interpreted as a quantitative measurement of the phase shift from the sample; otherwise the DPC image can only be used as a qualitative guide. Performing DPC below an absorption edge may mitigate this, depending on sample composition.

The measurements show that differential contrast can provide a complementary mechanism for spectro-microscopy which is readily collected in parallel with XRF-XANES. Complementary measurements can support investigations where XRF-XANES is potentially affected by non-linear effects from self-absorption or due to high count rates at strong white line features from thickness variation or higher concentration in a region of a sample under study.

The noise or uncertainty in the differential signal will define the sensitivity to a given edge step. This will depend, in part, on the angular sensitivity which depends on the distance from the sample to the detector and the noise, and the measurement sensitivity can be improved with longer sample–detector distances.

## 5. Conclusions

We have outlined data treatments to extract DPC and other contrast modes using a pixelated detector. Integration of the phase gradients using an anti-symmetric direction mirroring approach has been presented which results in phase images with a more uniform low-frequency background compared with standard mirroring approaches. The operation at beam focus for a DPC measurement provides computationally simple phase imaging at hard X-ray nano-focusing facilities, which can complement XRF measurements, and provide sufficient detail where only an identification of the structure of the light-element matrix is needed and resolution beyond the probe size is not required. DPC can provide quantitative information, and a comparison with ptychography shows that DPC phase images can compare favourably although with some compromises, such as spatial resolution. Scanning over an absorption edge for spectro-microscopy provides a measure of the total differential contrast which can be used as a complementary measurement to identify different chemical states. The quantification of the phase shift by the sample using DPC can only be directly interpreted when there is negligible absorption, and if this is not the case additional information is needed to separate the contributions of differential phase and differential absorption. This case is handled more effectively and more generally by ptychography.

Although the resolution of the DPC image is limited by the beam size, the simplicity of the measurement and phase-retrieval step can provide rapid feedback during experiments and produces good representative images of the sample, even with challenging, weakly scattering samples.
